# A Cross-Sectional Study on the Assessment of Service Quality and User Satisfaction of a Community Support Program in a Region of Taiwan

**DOI:** 10.3390/healthcare12131342

**Published:** 2024-07-05

**Authors:** Liang-Miin Tsai, Liang-Hsi Kung, Yu-Chen Tsai, Chih-Ming Kung, Yu-Hua Yan

**Affiliations:** 1 Tainan Municipal Hospital (Managed by Show Chwan Medical Care Corporation), Tainan 70173, Taiwan; 2lm658@mail.tmh.org.tw (L.-M.T.); 2u0635@mail.tmh.org.tw (L.-H.K.); 2t0101@mail.tmh.org.tw (Y.-C.T.); 2 Department of Information Technology and Communication, Shih Chien University Kaohsiung, Kaohsiung 84550, Taiwan; alex@cvig.org

**Keywords:** community support programs, patient satisfaction, quality of health care, community health services, personal autonomy, health services accessibility

## Abstract

This study aims to assess the service quality and user satisfaction of a community support program (CSP) in a specific administrative region of Taiwan. Employing a cross-sectional design, data were collected from 450 CSP users in the region via a questionnaire. Statistical analyses, including descriptive analysis, ANOVA, and Scheffe’s Test, were conducted using SPSS 22.0. The findings reveal that users aged 70–79 years with primary education, as well as those with demand or unknown demand for long-term care, reported the highest level of satisfaction with CSP services (mean = 4.5, SD = 0.7, *p* < 0.05). The study underscores the influence of user characteristics and their understanding of the services on satisfaction levels. These insights provide clear direction for policymakers in shaping the future of CSPs, emphasizing the importance of addressing user needs and enhancing awareness and the utilization of available services.

## 1. Introduction

Taiwan has experienced rapid aging, transitioning from an “aging society” to an “aged society” in just 25 years. This demographic shift highlighted significant challenges, particularly in the realm of long-term care services. Currently, Taiwan faces a critical shortage of long-term care personnel and infrastructure, posing substantial socio-political and economic implications [1,2].

To address the growing needs of its aging population, Taiwan has implemented various long-term care initiatives, including the Long-term Care 10-Year Project 2.0. Despite these efforts, coordination issues and inconsistent service quality have historically hindered the effectiveness of these programs [3,4]. As of recent reports from the Ministry of Health and Welfare, the utilization rates of long-term care services remain low, reflecting limited public awareness and understanding of available services [5].

The concept of “Aging in Place” has become central to Taiwan’s long-term care strategy, with initiatives like community support programs (CSPs) playing a pivotal role. Established under the auspices of the Long-term Care Service Network Project and the Long-term Care Policy 2.0, CSPs aim to enhance community-based care through facilities such as “Street-side Long-term Care Stops” and specialized centers for dementia care and disability deferment [6,7,8,9,10].

Despite international precedents in developed countries where community support programs are robustly integrated and highly utilized, Taiwan faces unique challenges. The quality and user satisfaction of CSPs in Taiwan have not been extensively studied, and the existing literature suggests that these programs are underutilized and inadequately understood by the public [11,12]. This gap underscores the need for research to evaluate and enhance the effectiveness of Taiwan’s CSPs within the broader context of long-term care services.

The Parasuraman, Zeithaml, and Berry (PZB) model stands as a recognized framework for assessing service quality, particularly in bridging gaps between customer expectations and actual service delivery [13,14,15,16]. This model’s relevance lies in its ability to pinpoint discrepancies that influence customer perceptions, with Gap 5—discrepancies between expected and perceived service—being pivotal. Utilizing the SERVQUAL instrument alongside the PZB model, this study seeks to explore CSP users’ perceptions and satisfaction levels in Taiwan. By identifying these gaps, the study aims to inform strategies to enhance service delivery and meet user expectations effectively.

This study aims to address the following question: What are the current levels of service quality and user satisfaction among CSP users in Taiwan, and how can these services be improved to better meet user expectations? The findings from this study are anticipated to contribute to the strengthening of regional long-term care services and facilitate the greater utilization of CSPs among individuals and families facing long-term care challenges.

## 2. Materials and Methods

### 2.1. Study Design and Participants

This research employed a cross-sectional study design to assess the satisfaction levels of CSP users in a specific administrative region of Taiwan. The study population consisted of individuals aged 50 years and above who had utilized at least one type of CSP service, such as meal delivery or health promotion activities, within a daycare setting. Data collection took place between January and March 2020 using a validated questionnaire distributed at CSP facilities. Participation was voluntary, and written informed consent was obtained from all participants.

### 2.2. Sample Size

Based on data indicating the widespread usage of CSP-related services by approximately 2,457,657 individuals [17], the minimum required sample size was calculated to be 384 using a formula for proportions. To ensure adequate statistical power and account for potential non-responses, the final sample size was set at 450 [18].

### 2.3. Questionnaire Design

This section explicitly presents the research objectives and their alignment with the Parasuraman, Zeithaml, and Berry (PZB) model. The study utilized the PZB model to assess the gap between CSP users’ expectations [19] and their actual experiences, employing SERVQUAL as the measurement tool.

### 2.4. Validity and Reliability of the Questionnaire

The initial 20-item questionnaire draft underwent content validity assessment by three experts in long-term care and CSP, achieving a content validity index (CVI) above 0.8 for all questions. Internal consistency reliability was confirmed with a Cronbach’s alpha exceeding 0.8 for all questionnaire facets.

### 2.5. Data Collection

Data were collected between October 2019 and March 2020 through the in-person administration of the questionnaire at CSP facilities. Trained researchers ensured uniformity and accuracy in data collection procedures.

### 2.6. Ethical Considerations

The study received ethical approval from the relevant institutional review board. Prior to participation, all respondents provided written informed consent, and assurances of confidentiality and anonymity of responses were maintained throughout the study.

### 2.7. Statistical Analysis

Regarding the statistical analysis, the term “*t*-test” was inaccurately used to refer to the exploration of influence. To clarify, *t*-tests were employed to assess significant differences between various groups, not to measure influence, impact, or effect sizes. The analysis aimed to determine whether there were statistically significant differences in satisfaction levels among CSP users based on demographic and service-related variables. Descriptive statistics, chi-square tests, and one-way analysis of variance (ANOVA) were also conducted to further explore these differences. Post hoc tests, specifically Scheffe’s test, were employed to identify specific group differences.

## 3. Results

### 3.1. Participants Characteristics

Table 1 presents detailed sociodemographic characteristics of the participants. The majority of participants were women (62.9%), predominantly aged 60 years and above (90%), with a significant proportion in their 70s (42.2%). Educational attainment was mostly primary school or junior high school (74.4%), and the majority of participants were married (66.9%). Financially, a large percentage reported well-off finances (88.9%), and they were primarily supported by pensions (47.8%) or assistance from children (28.2%). In terms of health status, 57.1% reported chronic diseases, while 58.7% did not have long-term care disability demands.

### 3.2. Degree of Satisfaction with CSP Services by User Traits

Table 2 illustrates the degree of satisfaction with CSP services across various user traits. Significant associations were found between education level and satisfaction in tangibles, reliability, responsiveness, empathy, and assurance. Participants with primary school education showed higher satisfaction compared to those with senior high school or college education. Marital status also influenced satisfaction, with divorced and widowed participants generally showing higher satisfaction with respect to tangibles. Age was significantly associated with higher satisfaction among those aged 70–79 years across all facets studied. There was variation in satisfaction based on long-term care disability demands, with those with unclear demands showing higher satisfaction in tangibles, responsiveness, empathy, and overall satisfaction, while those with demands scored higher in reliability and assurance.

### 3.3. Degree of Satisfaction with Services Provided by CSP

Table 3 outlines the degree of satisfaction with specific services provided by CSP. Awareness of services varied widely among participants, with higher awareness correlating significantly with higher overall satisfaction and satisfaction in tangibles, reliability, responsiveness, empathy, and assurance. Notably, health promotion activities were most widely known (78.4%), while awareness of other services such as care visiting, consultation and referral, and meal services was lower.

## 4. Discussion

### 4.1. Service Utilization and Demographic Trends

This study investigates the perceptions and satisfaction of CSP services among users, shedding light on significant associations with demographic characteristics. The predominance of female participants underscores the heightened demand for care services among women within Taiwan’s aging population [7]. Projections from the Taiwan Ministry of Health and Welfare suggest a demographic shift toward an increasing number of older females requiring long-term care, necessitating a nuanced approach to service design [20].

### 4.2. Factors Influencing Satisfaction

The study observed significant associations between education level and satisfaction, with participants having lower educational attainment expressing higher satisfaction. This phenomenon may stem from more modest expectations and a greater appreciation for the supportive services provided by CSP. Financially, the reliance on pensions and familial support aligns with national trends among older adults, highlighting economic vulnerability exacerbated by changing family dynamics and living patterns [21,22].

Regarding health status, a notable proportion of participants reported uncertainty about their long-term care needs, reflecting broader challenges in disability awareness and readiness for aging-in-place initiatives. Chronic disease prevalence was high among our participants, mirroring national trends of increasing health complexities in an aging society. CSP’s provision of professional medical consultation and referral services emerges as crucial in managing chronic conditions and preventing further health deterioration [23,24,25].

### 4.3. Satisfaction with CSP Services

Participants expressed overall satisfaction with CSP services across tangibles, empathy, responsiveness, assurance, and reliability dimensions, with tangibles receiving the highest scores. This contrasts with some previous findings, suggesting that comprehensive service offerings outweigh the influence of physical facility conditions on satisfaction levels [6,26]. The nuanced understanding of CSP’s diverse service components significantly correlated with higher satisfaction levels among users, underscoring the role of service comprehensibility in enhancing user experience and satisfaction.

### 4.4. Future Implications

CSP services encompass a wide array of offerings, including care visits, consultations, meals, health promotion, long-term care information, medical consultations, and volunteer services. The study identifies a direct relationship between users’ understanding of these services and their satisfaction levels. Tailoring services to individual needs emerges as pivotal in promoting active aging and enhancing the quality of life among seniors [27,28].

## 5. Limitations and Future Directions

This study is limited by its geographical focus on a specific administrative region of Taiwan and its cross-sectional design. Future research should consider longitudinal approaches and broader geographical coverage to enhance generalizability. Insights gained from this study inform potential enhancements in service delivery and policy frameworks aimed at effectively supporting Taiwan’s aging population.

## 6. Conclusions

This study underscores the critical importance of community support programs (CSPs) in addressing the complex needs of Taiwan’s aging population. Through the use of the SERVQUAL questionnaire, the research reveals a significant correlation between users’ understanding of CSP services and their satisfaction levels.

The findings highlight the indispensable role of CSPs in enhancing the quality of life for seniors and supporting their families. By integrating these services into broader healthcare frameworks, Taiwan can effectively meet the challenges of an aging society. This study advocates for continued investment in CSPs to ensure they remain integral to Taiwan’s healthcare strategy.

In conclusion, this research provides compelling evidence that reinforces the need for sustained support and development of CSPs, emphasizing their pivotal role in promoting active aging and addressing long-term care challenges in Taiwan.

## Figures and Tables

**Table 1:** Characteristics of the study population (n = 450).

Variable		Total n	%
Gender			
	Female	283	62.9
	Male	167	37.1
Education level			
	Primary school	203	45.1
	Junior high school	132	29.3
	Senior high school	71	15.8
	Associate degree	29	6.4
	Bachelor’s degree	15	3.3
Marital status			
	Single	17	3.8
	Married	301	66.9
	Divorced	18	4.0
	Widowed	114	25.3
Age (years)			
	<60	46	10.2
	60–69	138	30.7
	70–79	190	42.2
	>80	76	16.9
Economic status			
	Difficult	42	9.3
	Well-off	400	88.9
	Rich	8	1.8
Financial resources			
	Children give	127	28.2
	Government subsidy	49	10.9
	Labor service	59	13.1
	Pension	215	47.8
Long-term care disabled demand			
	No	264	58.7
	Yes	186	41.3
Chronic diseases			
	Yes	257	57.1
	No	193	42.9

**Table 2:** Difference in CBCC degree of satisfaction regarding users’ traits.

Item		Tangibles	Reliability	Responsiveness	Empathy	Assurance	Overall Satisfaction
Gender:							
	Female	4.37	4.21	4.24	4.15	4.28	4.26
	Male	4.39	4.17	4.30	4.14	4.32	4.27
	*p* value	0.461	0.864	0.568	0.915	0.608	0.843
Education level:							
	Primary school (1)	4.56	4.39	4.47	4.37	4.49	4.46
	Junior high school (2)	4.41	4.26	4.32	4.21	4.35	4.31
	Senior high school (3)	4.06	3.81	3.84	3.66	3.92	3.87
	Associate degree (4)	4.17	3.95	4.10	3.98	4.12	4.07
	Bachelor’s degree (5)	3.47	3.337	3.37	3.37	3.40	3.39
	*p* value	<0.001	<0.001	<0.001	<0.001	<0.001	<0.001
	Scheffe’s	1 > 3, 5	1 > 3, 5	1 > 3, 5	1 > 3, 5	1 > 3, 5	1 > 3, 5
Marital status:							
	Single (1)	3.98	4.00	3.94	3.91	3.98	3.97
	Married (2)	4.31	4.10	4.18	4.05	4.23	4.18
	Divorced (3)	4.69	4.50	4.53	4.50	4.63	4.58
	Widowed (4)	4.55	4.43	4.49	4.39	4.47	4.47
	*p* value	<0.001	<0.001	<0.001	<0.001	0.002	<0.001
	Scheffe’s	3 > 1, 4 > 1	4 > 2	4 > 2	4 > 2	4 > 2	4 > 2
Age (years):							
	<60(1)	4.06	3.88	3.88	3.76	3.99	3.93
	60–69(2)	4.36	4.18	4.24	4.12	4.26	4.24
	70–79(3)	4.51	4.31	4.41	4.29	4.42	4.40
	>80(4)	4.27	4.12	4.18	4.09	4.22	4.18
	*p* value	<0.001	0.006	<0.000	<0.001	0.004	<0.001
	Scheffe’s	3 > 1	3 > 1	3 > 1	3 > 1	3 > 1	3 > 1
Economic status:							
	Difficult	4.37	4.33	4.28	4.23	4.3	4.32
	Well-off	4.37	4.17	4.26	4.13	4.29	4.25
	Rich	4.75	4.54	4.50	4.50	4.58	4.59
	*p* value	0.336	0.203	0.665	0.385	0.552	0.395
Long-term care disabled demand:							
	No	4.26	4.12	4.15	4.05	4.19	4.17
	Yes	4.53	4.29	4.42	4.28	4.44	4.44
	*p* value	<0.001	0.016	<0.001	0.003	<0.001	<0.001
Chronic diseases:							
	Yes	4.41	4.20	4.29	4.17	4.33	4.29
	No	4.33	4.19	4.23	4.12	4.25	4.23
	*p* value	0.246	0.896	0.433	0.589	0.231	0.420

Note: If there is no significant difference, no ex post verification will be conducted.

**Table 3:** Degree of satisfaction difference analysis on services provided by CBCCs (*t*-test).

Item		Tangibles	Reliability	Responsiveness	Empathy	Assurance	Overall Satisfaction
Understanding of care visiting services provided by CBCCs							
	Yes (n = 114)	4.42	4.24	4.31	4.19	4.34	4.31
	No (n = 336)	3.67	3.44	3.48	3.40	3.53	3.51
	*p* value	<0.001	<0.001	<0.001	<0.001	<0.001	<0.001
Understanding of consultation and referral service by CBCCs							
	Yes (n = 128)	4.44	4.27	4.33	4.22	4.36	4.33
	No (n = 322)	3.57	3.29	3.38	3.29	3.42	3.40
	*p* value	<0.001	<0.001	<0.001	<0.001	<0.001	<0.001
Understanding of meal service by CBCCs							
	Yes (n = 108)	4.45	4.27	4.35	4.23	4.37	4.34
	No (n = 342)	3.37	3.20	3.23	3.14	3.33	3.27
	*p* value	<0.001	<0.001	<0.001	<0.001	<0.001	<0.001
Understanding of health promotion activities by CBCCs							
	Yes (n = 353)	4.42	4.24	4.31	4.19	4.34	4.31
	No (n = 97)	3.44	3.32	3.31	3.24	3.38	3.35
	*p* value	<0.001	<0.001	<0.001	<0.001	<0.001	<0.001
Understanding of long-term care information by CBCCs							
	Yes (n = 147)	4.43	4.25	4.32	4.21	4.36	4.32
	No (n = 303)	3.91	3.69	3.72	3.59	3.71	3.74
	*p* value	<0.001	<0.001	<0.001	<0.001	<0.001	<0.001
Understanding of medical and care-related consultation services by CBCCs							
	Yes (n = 155)	4.44	4.27	4.34	4.23	4.37	4.37
	No (n = 295)	3.93	3.67	3.76	3.62	3.78	3.76
	*p* value	<0.001	<0.001	<0.001	<0.001	<0.001	<0.001
Understanding of volunteer service by CBCCs							
	Yes (n = 220)	4.41	4.26	4.31	4.21	4.34	4.32
	No (n = 230)	4.23	3.913	4.06	3.90	4.09	4.05
	*p* value	0.031	<0.001	0.007	0.002	0.007	0.002

## Data Availability

Data cannot be made publicly available owing to the fact that the privacy of individual participants cannot be compromised. However, the dataset is available from the corresponding author upon reasonable request.
